# Characterization of the *MLO* gene family in Rosaceae and gene expression analysis in *Malus domestica*

**DOI:** 10.1186/1471-2164-15-618

**Published:** 2014-07-22

**Authors:** Stefano Pessina, Stefano Pavan, Domenico Catalano, Alessandra Gallotta, Richard GF Visser, Yuling Bai, Mickael Malnoy, Henk J Schouten

**Affiliations:** Department of Genomics and Biology of Fruit Crops, Fondazione Edmund Mach, via E. Mach 1, 38010 San Michele all’Adige, Italy; Wageningen UR Plant Breeding, Wageningen University and Research Centre, P.O Box 16, 6700 AA Wageningen, The Netherlands; Department of Soil, Plant and Food Science, University of Bari, Via Amendola 165/A, 70126 Bari, Italy; National Research Council, Institute of Plant Genetics, Via Amendola 165/A, 70126 Bari, Italy

**Keywords:** Rosaceae, MLO, Powdery mildew, *Malus domestica*

## Abstract

**Background:**

Powdery mildew (PM) is a major fungal disease of thousands of plant species, including many cultivated Rosaceae. PM pathogenesis is associated with up-regulation of *MLO* genes during early stages of infection, causing down-regulation of plant defense pathways. Specific members of the *MLO* gene family act as PM-susceptibility genes, as their loss-of-function mutations grant durable and broad-spectrum resistance.

**Results:**

We carried out a genome-wide characterization of the *MLO* gene family in apple, peach and strawberry, and we isolated apricot *MLO* homologs through a PCR-approach. Evolutionary relationships between *MLO* homologs were studied and syntenic blocks constructed. Homologs that are candidates for being PM susceptibility genes were inferred by phylogenetic relationships with functionally characterized *MLO* genes and, in apple, by monitoring their expression following inoculation with the PM causal pathogen *Podosphaera leucotricha.*

**Conclusions:**

Genomic tools available for Rosaceae were exploited in order to characterize the *MLO* gene family. Candidate *MLO* susceptibility genes were identified. In follow-up studies it can be investigated whether silencing or a loss-of-function mutations in one or more of these candidate genes leads to PM resistance.

**Electronic supplementary material:**

The online version of this article (doi:10.1186/1471-2164-15-618) contains supplementary material, which is available to authorized users.

## Background

Powdery mildew (PM) is a major fungal disease for thousands of plant species [1], including cultivated Rosaceae such as apple (*Malus domestica)*, apricot (*Prunus armeniaca),* peach (*Prunus persica),* and strawberry (*Fragaria x ananassa)*. Powdery mildew occurs in all major growing regions of Rosaceous crops, leading to severe losses [2] *.* The major PM causal agents are *Podosphaera leucotricha* in apple [2], *Sphaerotheca pannosa* var. *persicae* in peach [3], *Podosphaera tridactyla* in apricot [4] and *Podosphaera aphanis* (syn. *Sphaerotheca macularis* f. sp. *fragariae)* in strawberry [5]. Powdery mildew shows similar symptoms in the four species: white spots appear on young green tissues, particularly leaves in the first days after opening, whereas mature leaves show some resistance. Infected leaves crinkle, curl, and prematurely drop. Blossoms and fruits are not the primary targets of PM fungi, but infections of these tissues are possible [2, 3, 5]. In peach, apricot and apple, PM spores overwinter in buds and then in spring, with the reprise of vegetative growth, the spores start a new infection [2, 3].

Cultivars resistant to PM are fundamental in order to reduce the use of pesticides in agricultural practices. The usual strategy in breeding focuses on dominant plant resistance genes (R-genes), however these genes often originate from wild-relatives of the cultivated species, and thus interspecific crossability barriers could prevent their introgression [6]. Moreover, in case of a successful cross, several undesirable traits are incorporated with the R-gene, making extensive backcrossing necessary, which is time-consuming in woody species. Finally, the durability of R-genes is generally limited due to the appearance of virulent strains of the pathogen, which can overcome resistance in a few years [7]. Two examples are *Venturia inaequalis* race 6, which is able to overcome *Rvi6* resistance to scab in apple [8], and *P. leucotricha* strains able to breakdown *Pl-1* and *Pl-2*, two major PM R-genes of apple [9].

An alternative to the use of R-genes is based on plant susceptibility genes (S-genes), defined as genes whose loss-of-function results in recessively inherited resistance [10]. Barley *mlo* PM resistance, first characterized in 1942, is a remarkable example of immunity due to the absence of an S-gene, as it derives from a loss-of-function mutation in the *MLO* (***M****ildew****L****ocus****O***) gene, encoding for a protein with seven transmembrane domains [11, 12]. *mlo* resistance has long been considered as a unique form of immunity, characterized by durability, broad-spectrum effectiveness and recessive inheritance [13]. However*,* the characterization of the sources of resistance in other plant species, such as *Arabidopsis* [14], pea [15, 16] and tomato [17], has revealed that resistance resulting from loss-of-function mutations in *MLO* functional orthologs is more common than previously thought. Therefore, it has been suggested that the inactivation of *MLO* susceptibility genes could represent a valid strategy to introduce PM resistance across a broad range of cultivated species [10].

Histological characterization of *mlo* resistance revealed that it is based on a pre-penetration defense system, associated with the formation of cell-wall appositions [14, 18] and at least partially dependent on the actin cytoskeleton [19]. It has been suggested that functional MLO proteins negatively regulate vesicle-associated and actin-dependent defense pathways at the site of attempted PM penetration [20]. MLO proteins are therefore targeted by PM fungi as a strategy to induce pathogenesis. The early stages of PM infection are associated with an increase in transcript abundance of *MLO* susceptibility genes, showing a peak at six hours after inoculation. This has been shown to occur in tomato [17], barley [21], pepper [22] and grape [23, 24].

*MLO* susceptibility genes are members of a gene family which shows tissue specific expression patterns and are involved in a variety of physiological processes, besides the response to PM fungi: one of the 15 *MLO* genes of *Arabidopsis, AtMLO7,* is involved in pollen tube reception by the embryo sac and its mutation results in reduced fertility [25]. Two other *Arabidopsis* genes, named *AtMLO4* and *AtMLO11,* are involved in the control of root architecture, as mutants with null alleles of these two genes display asymmetrical root growth and exaggerated curvature [26].

Previous phylogenetic analysis of the MLO protein family identified six clades [23]. Currently, all MLO proteins functionally related to PM susceptibility in dicot species appear in a single clade, namely Clade V [14, 17, 23, 24]. Similarly, Clade IV harbours all characterized PM susceptibility proteins from monocots [20, 27].

*MLO* genes have been intensively studied in many monocots and dicots, but very little has been performed in Rosaceae. In this investigation, we characterized the *MLO* gene family in a number of Rosaceous species, with respect to their structural, genomic and evolutionary features. Moreover, we monitored the transcript abundances of apple *MLO* homologs following *P. leucotricha* inoculation in three apple cultivars.

## Results

### *In silico*and *in vitro*characterization of Rosaceae *MLO*homologs

A database search for Rosaceae *MLO* homologs produced 21 significant matches in peach, 23 in strawberry and 28 in apple. Of these, six (five from *M. domestica* and one from *F. vesca*) showed a very limited alignment region with other *MLO* genes, whereas eight (two from *M. domestica*, two from *P. persica* and four from *F. vesca*) were characterized by markedly different length with respect to *MLO* homologs reported in the genomes of *Arabidopsis* and grapevine [23, 28], i.e. less than 350 amino acids (aa) or more than 700 aa. Details on genomic localization amino acid number, putative transmembrane domains and predicted exon/intron structure of the remaining homologs, together with information about the *MLO* homologs nomenclature chosen in this study is provided in Tables 1, 2 and 3.

**Table 1:** **Members of the** ***MdMLO*** **gene family as predicted in** ***M. domestica*** **cv. Golden Delicious genome sequence**

Gene	Accession number ^a^	Chr.	Starting position (Mb)	Clade	Introns	TM ^b^	Amino acids	Conserved aa ^c^
*MdMLO1*	MDP0000177099	2	1.02	II	11	3	487	25
*MdMLO2*	MDP0000240125	2	11.10	I	11	3	571	20
*MdMLO3*	MDP0000168575	2	11.11	I	13	7	670	22
*MdMLO4*	MDP0000207002	2	8.79	III	16	7	634	28
*MdMLO5*	MDP0000163089	9	15.26	V	14	6	579	30
*MdMLO6*	MDP0000119433	3	33.95	II	0	7	504	30
*MdMLO7*	MDP0000123907	n.d.	n.d.	V	n.d.	6	561	28
*MdMLO8*	MDP0000218520	2	11.11	I	9	4	390	14
*MdMLO9*	MDP0000320797	2	27.20	II	10	5	454	28
*MdMLO10*	MDP0000196373	3	26.97	I	13	6	539	28
*MdMLO11*	MDP0000239643	4	9.84	V	12	8	575	28
*MdMLO12*	MDP0000133162	6	0.81	III	13	5	516	28
*MdMLO13*	MDP0000142608	7	7.48	II	12	6	351	18
*MdMLO14*	MDP0000191469	8	29.25	II	10	5	395	23
*MdMLO15*	MDP0000141595	9	7.54	III	15	6	647	24
*MdMLO16*	MDP0000191848	9	21.12	VI	14	6	606	29
*MdMLO17*	MDP0000145097	11	27.97	I	13	7	523	28
*MdMLO18*	MDP0000928368	10	27.97	VII	12	7	502	30
*MdMLO19*	MDP0000168714	12	16.23	V	13	7	590	30
*MdMLO20*	MDP0000134649	13	11.61	VIII	13	5	589	27
*MdMLO21*	MDP0000133760	15	24.99	VI	15	6	560	28

^a^Available at http://www.rosaceae.org/gb/gbrowse/malus_x_domestica/.

^b^Number of transmembrane domains in the predicted protein, as determined by InterPro prediction software (http://www.ebi.ac.uk/interpro/).

^c^number of conserved amino acids out of the 30 identified by Elliot *et al.* [29].

**Table 2:** **Members of the** ***PpMLO*** **gene family as predicted in** ***Prunus persica*** **genome sequence**

Gene	Accession number ^a^	Chr.	Starting position (Mb)	Clade	Introns	TM ^b^	Amino acids	Conserved aa ^c^
*PpMLO1*	ppa003207m	6	6.82	V	14	7	593	30
*PpMLO2*	ppa003435m	7	18.38	III	14	8	574	30
*PpMLO3*	ppa003437m	6	21.99	V	13	7	574	30
*PpMLO4*	ppa003466m	2	21.03	V	14	7	572	30
*PpMLO5*	ppa003706m	4	10.92	I	14	8	555	30
*PpMLO6*	ppa004012m	7	22.64	II	14	6	535	29
*PpMLO7*	ppa004508m	8	21.17	II	0	7	506	29
*PpMLO8*	ppa004621m	6	22.01	VI	14	6	499	29
*PpMLO9*	ppa004687m	4	2.59	VII	11	7	496	29
*PpMLO10*	ppa004866m	2	13.73	II	11	7	488	29
*PpMLO11*	ppa020172m	1	43.04	I	14	4	561	30
*PpMLO12*	ppa020311m	5	0.82	IV	13	7	566	30
*PpMLO13*	ppa021048m	4	15.57	VIII	12	5	510	24
*PpMLO14*	ppa022847m	6	6.80	VI	14	6	550	29
*PpMLO15*	ppa024476m	7	17.63	I	14	8	539	26
*PpMLO16*	ppa024488m	5	0.76	III	14	6	504	30
*PpMLO17*	ppa024581m	6	8.95	II	13	6	463	27
*PpMLO18*	ppa026565m	6	22.00	VI	13	6	416	25
*PpMLO19*	ppb024523m	1	42.04	II	13	5	446	23

^a^Available at http://www.rosaceae.org/gb/gbrowse/prunus_persica/.

^b^Number of transmembrane domains in the predicted protein, as determined by InterPro prediction software (http://www.ebi.ac.uk/interpro/).

^c^number of conserved amino acids out of the 30 identified by Elliot *et al.* [29].

**Table 3:** **Members of the** ***FvMLO*** **gene family as predicted in** ***Fragaria vesca*** **genome sequence**

Gene	Accession number ^a^	Chr.	Starting position (Mb)	Clade	Introns	TM ^b^	Amino acids	Conserved aa ^c^
*FvMLO1*	mrna02774.1-v1.0-hybrid	n.d.	n.d.	V	14	7	632	28
*FvMLO2*	mrna03210.1-v1.0-hybrid	3	14.46	II	11	5	528	20
*FvMLO3*	mrna09651.1-v1.0-hybrid	6	35.88	III	14	6	542	28
*FvMLO4*	mrna09653.1-v1.0-hybrid	6	35.90	V	14	7	573	30
*FvMLO5*	mrna10166.1-v1.0-hybrid	1	1.34	II	14	3	688	26
*FvMLO6*	mrna10346.1-v1.0-hybrid	3	12.52	II	7	2	385	15
*FvMLO7*	mrna10363.1-v1.0-hybrid	3	12.49	II	9	2	442	21
*FvMLO8*	mrna10558.1-v1.0-hybrid	2	19.08	II	n.d.	6	514	28
*FvMLO9*	mrna11028.1-v1.0-hybrid	n.d.	n.a.	I	10	4	434	18
*FvMLO10*	mrna13023.1-v1.0-hybrid	1	7.96	III	13	6	557	27
*FvMLO11*	mrna14592.1-v1.0-hybrid	1	8.77	I	13	7	548	28
*FvMLO12*	mrna23198.1-v1.0-hybrid	7	15.89	V	14	7	507	29
*FvMLO13*	mrna26428.1-v1.0-hybrid	7	17,79	VIII	11	5	558	20
*FvMLO14*	mrna28541.1-v1.0-hybrid	n.d.	n.a.	III	11	4	481	26
*FvMLO15*	mrna29770.1-v1.0-hybrid	3	7.36	VII	13	7	538	28
*FvMLO16*	mrna31264.1-v1.0-hybrid	3	30.51	I	16	8	579	28
*FvMLO17*	mrna31498.1-v1.0-hybrid	5	20.23	IV	11	5	531	27
*FvMLO18*	mrna29285.1-v1.0-hybrid	5	19.12	V	6	4	357	18

^a^Available at http://www.rosaceae.org/gb/gbrowse/fragaria_vesca_v1.0-lg/ (hybrid).

^b^Number of transmembrane domains in the predicted protein, as determined by InterPro prediction software (http://www.ebi.ac.uk/interpro/).

^c^number of conserved amino acids out of the 30 identified by Elliot *et al.* [29].

Peach and apricot are evolutionary very close to each other, and show a high degree of homology in DNA sequence. Phylogenetic analysis (see next paragraph) indicated peach homologs *PpMLO1*, *PpMLO3* and *PpMLO4* as candidates for being required for PM susceptibility. Therefore, we used the sequences of these genes to design primers to identify full-length apricot *MLO* genes. This approach resulted in the amplification and the successive characterization of three *MLO* sequences, which were by analogy named *PaMLO1, PaMLO3,* and *PaMLO4* (deposited in the NCBI database with the accession numbers KF177395, KF177396, and KF177397, respectively).

### Phylogenetic relations and inference of orthology

**Figure 1:**
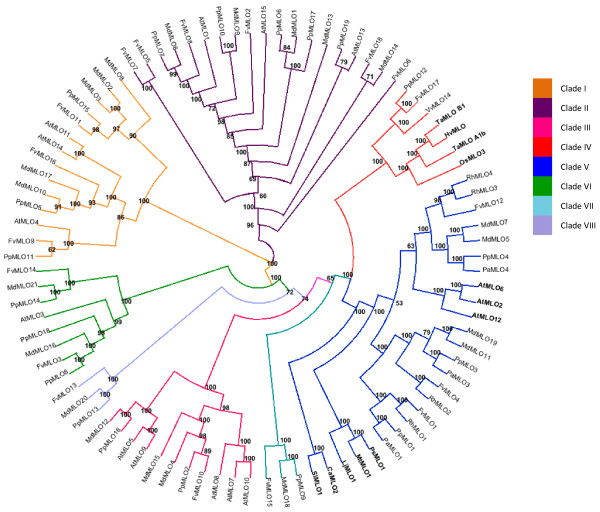
**Phylogenetic tree of Rosaceae MLO.** Phylogenetic relationships of predicted Rosaceae MLO amino acid sequences to MLO proteins of other plant species. The dataset includes Rosaceae MLO sequences from *Rosa hybrida* (RhMLO), *Malus domestica* (MdMLO), *Prunus persica* (PpMLO), *Prunus armeniaca* (PaMLO) and *Fragaria vesca* (FvMLO). The other proteins included are *Solanum lycopersicum* SlMLO1, *Arabidopsis thaliana* AtMLO, *Capsicum annuum* CaMLO2, *Pisum sativum* PsMLO1, *Medicago truncatula* MtMLO1, *Lotus japonicus* LjMLO1, *Vitis vinifera* VvMLO14, *Hordeum vulgare* HvMLO, *Triticum aestivum* TaMLO_B1*,* TaMLO_A1b and *Oryza sativa* OsMLO2. Proteins which have been functionally characterized as susceptibility genes are highlighted in bold. Numbers at each node represent bootstrap support values (out of 100 replicates).

We performed a phylogenetic study on the newly identified Rosaceae MLO proteins. The dataset was completed with four homologs recently characterized in *Rosa hybrida* (rose) [30] (RhMLO1, RhMLO2, RhMLO3 and RhMLO4), the complete *Arabidopsis thaliana* AtMLO protein family [14], a series of MLO homologs which have been functionally associated with PM susceptibility, namely tomato (*Solanum lycopersicum*) SlMLO1 [17], pea (*Pisum sativum*) PsMLO1 [15, 16], pepper (*Capsicum annuum*) CaMLO2 [27], lotus (*Lotus japonicus*) LjMLO1 [15], barrel clover (*Medicago truncatula*) MtMLO1 [15], barley (*Hordeum vulgare*) HvMLO [11], rice (*Oryza sativa*) OsMLO2 [31], wheat (*Triticum aestivum*) TaMLO_B1 and TaMLO_A1b [31], and grapevine (*Vitis vinifera*) VvMLO14, the only dicot MLO homolog known to belong to clade IV [23]. Clustering analysis using the UPGMA algorithm resulted in a total of eight distinct clades and no divergent lineage (Figure 1). Clade numbers from I to VI were assigned based on the position of *Arabidopsis* AtMLO homologs and barley HvMLO, according to the previous study of Feechan *et al.* [23]. The two additional clades (named VII and VIII) were found to include Rosaceae MLO homologs only, both having one homolog from *P. persica*, one from *F. vesca* and one from *M. domestica*. Further clustering analysis with a Neighbour-Joining algorithm resulted in merging clade VII and VIII (not shown).

Four apple MLO homologs (MdMLO5, MdMLO7, MdMLO11 and MdMLO19) and three MLO homologs from peach (PpMLO1, PpMLO3 and PpMLO4), apricot (PaMLO1, PaMLO3 and PaMLO4) and woodland strawberrry (FvMLO1, FvMLO4 and FvMLO12) were found to cluster together in the phylogenetic clade V, containing all the dicot MLO proteins experimentally shown to be required for PM susceptibility (e.g. [16, 23]. One homolog from strawberry (FvMLO17) and one from peach (PpMLO12) were found to group, together with grapevine VvMLO14, in clade IV, which contains all monocot MLO proteins acting as PM susceptibility factors (Figure 1).

**Figure 2:**
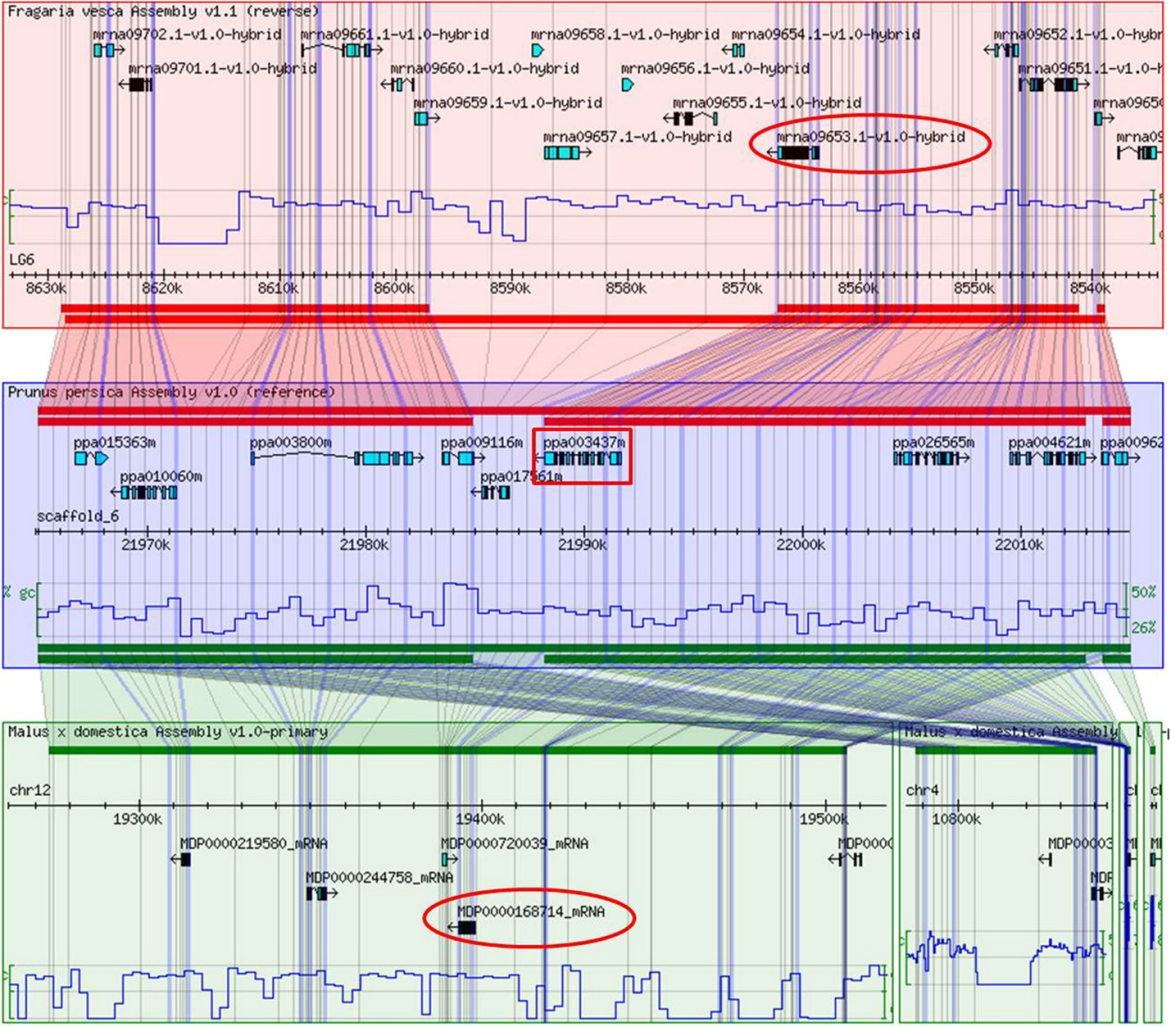
**Synteny between apple, peach and strawberry.** Results of search for *F. vesca* and *M. domestica* chromosomal regions syntenic to a *P. persica* 50 kb stretch including the *MLO* homolog *PpMLO3* (corresponding to ppa003437m in the genomic database of Rosaceae), boxed. Shaded polygons indicate aligned regions between genomes. Grid lines are meant to indicate insertions/deletions between the genomes of *F. vesca* and *M. domestica* with respect to the *P. persica* reference sequence. Strawberry *FvMLO4* and apple *MdMLO19* (named in the figure as mrna09653.1-v1.0-hybrid and MDP0000168714, according to the nomenclature provided in this paper), predicted to be *PpMLO3* orthologs, are indicated with circles.

We used the GBrowse-Syn tool to detect syntenic blocks encompassing *P. persica*, *F. vesca* and *M. domestica MLO* genes. As syntenic blocks derive from the evolution of the same chromosomal region after speciation, orthology between *MLO* genes could be inferred. In total, twelve orthologous relationships were predicted between *P. persica* and *F. vesca*, nine between *P. persica* and *M. domestica* and eight between *F. vesca* and *M. domestica* (Table 4, Figure 2 and Additional file 1).

**Table 4:** **Relations of orthology inferred between** ***P. persica, F. vesca*** **and** ***M. domestica MLO*** **homologs**

***P. persica***genes	***F. vesca***orthologs	***M. domestica***orthologs
*PpMLO2*	*FvMLO10*	*MdMLO15*
*PpMLO3*	*FvMLO4*	*MdMLO19*
*PpMLO4*	*FvMLO12*	*-*
*PpMLO5*	*FvMLO16*	*MdMLO10, MdMLO17*
*PpMLO6*	*FvMLO5*	*MdMLO1*
*PpMLO7*	*FvMLO8*	*-*
*PpMLO8*	*FvMLO3*	*-*
*PpMLO9*	*FvMLO15*	*MdMLO18*
*PpMLO10*	*FvMLO2*	*MdMLO9*
*PpMLO14*	*FvMLO14*	*MdMLO21*
*PpMLO15*	*FvMLO11*	*-*
*PpMLO16*	-	*MdMLO12*
*PpMLO18*	*FvMLO3*	*-*

Relations of orthology between *PpMLO1, PpMLO3*, *PpMLO4* and apricot *PaMLO1, PaMLO3*, *PaMLO4* were clearly suggested by the high percentage of sequence identity between these homolog genes, which was 97,3%, 98,8% and 96,7%, respectively.

### Transcription of putative apple *MLO*genes in response to *Podosphaera leucotricha*inoculation

**Figure 3:**
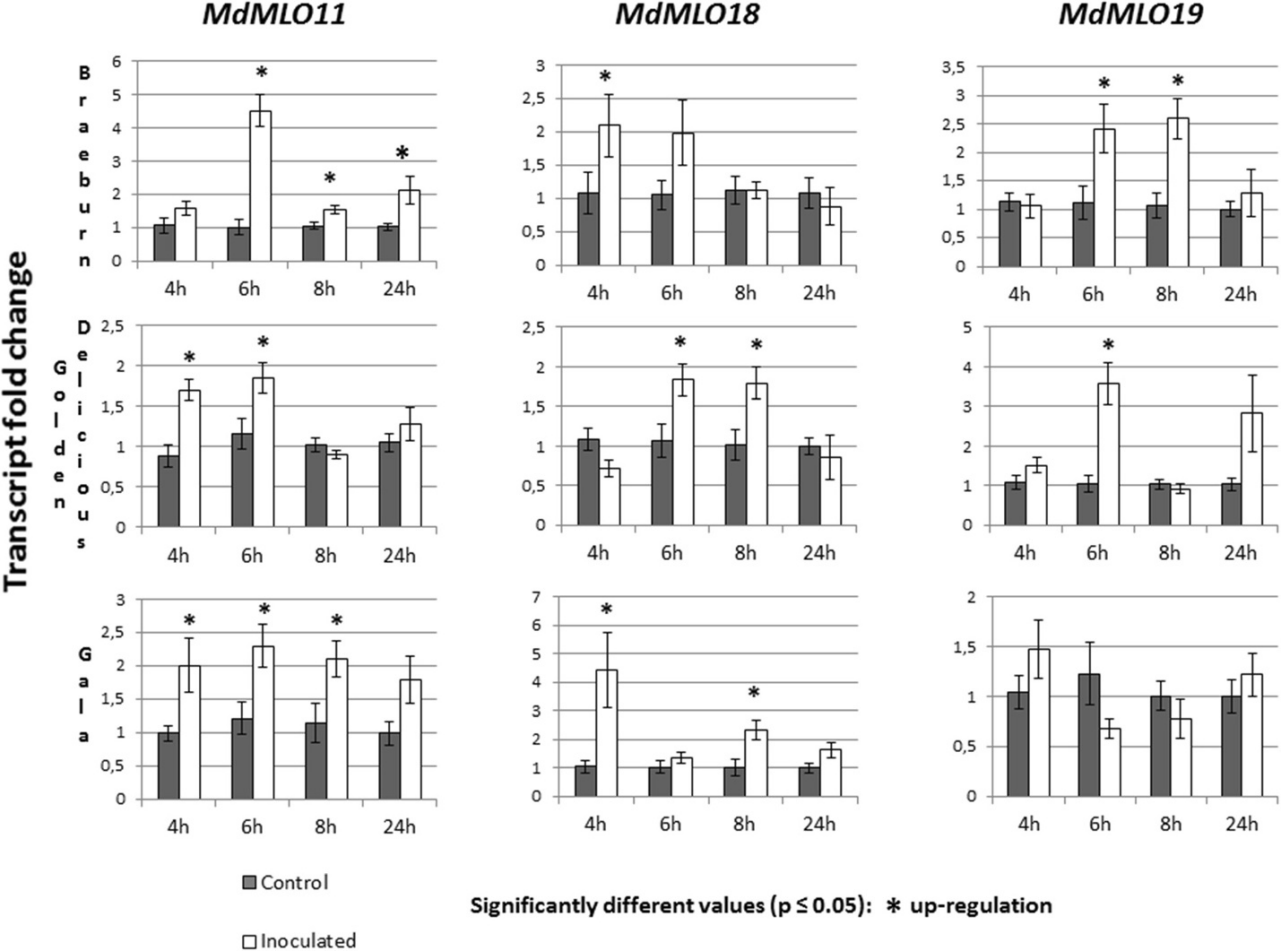
**Transcriptional variation of three apple MLO genes following inoculation with** ***P. leucotricha.*** Transcript abundances of three *MLO* genes in leaves of the apple cultivars ‘Braeburn’, ‘Golden Delicious’ and ‘Gala’, following powdery mildew (PM) inoculation. Here we show only *MLO* genes that were more than one time significantly up or down regulated following PM inoculation at one of the four time points examined (4, 6, 8 and 24 hpi). The set of results of all investigated genes is shown in Additional file 1: Figure S1. Each bar shows the average of four to eight biological replicates. The Ct values have been normalized for three reference genes: actin, ubiquitin and elongation factor 1. Statistical significance was determined with a *t*-test for each individual pair of inoculated and non-inoculated samples at each time point. The error bars show standard errors of the means. Significant differences between inoculated samples and control samples are indicated with an asterisk (*P* < 0.05).

To identify *MLO* genes that respond to the PM fungus *P. leucotricha*, we measured the transcript abundance of 19 out of 21 apple *MLO* genes in leaves 4, 6, 8 and 24 hours after artificial inoculation with the pathogen, and compared these data with the ones of non-inoculated leaves. Three cultivars, Golden Delicious, Braeburn and Gala, were analysed in order to investigate whether up-regulation was comparable among them and results could therefore be generalized for all apple cultivars. Three genes, namely *MdMLO11*, *MdMLO18* and *MdMLO19*, were found to be significantly up-regulated after inoculation with the pathogen (Figure 3 and Additional file 2). Up-regulation of these genes was about 2-fold compared to non-inoculated plants, with peaks of 4-fold up-regulation at very early time points (‘Braeburn’- *MdMLO11* - 6 hpi; ‘Gala’- *MdMLO18* - 4 hpi; ‘Golden Delicious’- *MdMLO19* - 6hpi). *MdMLO11* and *MdMLO18* were up-regulated in all cultivars, while *MdMLO19* was only up-regulated in ‘Braeburn’ and ‘Golden Delicious’.

Two of the genes, *MdMLO11* and *MdMLO19* belong to Clade V, while *MdMLO18* belongs to the newly identified Clade VII (Figure 1).

## Discussion

### Genomic organization and phylogenetic relations between Rosaceae *MLO*homologs

We report here the identification, through an *in silico* approach, of 19 *MLO* homologs in the genome of peach and 18 in the genome of strawberry. This is consistent with the results of previous genome-wide studies carried out on dicotyledonous species, indicating the presence of 15 *MLO* homologs in *Arabidopsis*, 17 in grapevine and 16 in tomato [9, 13]; Appiano *et al.*, unpublished results; [24]. Conversely, the number of *MLO* homologs detected in apple (21) is lower than expected, considering that a relatively recent genome-wide duplication event has occurred in the Pyreae tribe [32].

Most *PpMLO, FvMLO* and *MdMLO* homologs appeared to be widely distributed within the respective genomes (Tables 1, 2 and 3), indicating segmental duplication as the prevailing evolutionary mechanism for the Rosaceae *MLO* gene family. However, we also found cases of clusters of adjacent homologs (*PpMLO3, PpMLO8* and *PpMLO18, PpMLO12 and PpMLO16, PpMLO1* and *PpMLO14, FvMLO3* and *FvMLO4, FvMLO6* and *FvMLO7, MdMLO2, MdMLO3* and *MdMLO8*), which are likely the result of tandem duplication events.

Inference of phylogenetic relationships between MLO proteins revealed the presence of apple, strawberry, peach and apricot homologs in the clade V, containing all dicot MLO homologs shown so far to be involved in PM susceptibility, thus making them candidates to act as susceptibility factors. Although the simple clustering in clade V is not enough to recognize a gene as a susceptibility factor, it does provide the first evidence for functionality and allows for the reduction in the number of candidates for further functional analysis. Clade IV, that contains functional MLO susceptibility homologs from monocots, was found to include one homolog from *F. vesca* (FvMLO17) and one from *P. persica* (PpMLO12). In accordance with this finding, a MLO homolog from the dicot species *V. vinifera* also clusters in clade IV [23, 24]. Figure 1). Interestingly, phylogenetic analyses carried out in this study also revealed the presence of one or two additional clades, depending on the type of phylogenetic reconstruction (UPGMA or Neighbour-Joining), which were not reported to occur in earlier investigations. Moreover, they appear to be characteristic of Rosaceae, since they contain only homologs from this family. Clearly, the exclusivity for Rosaceae of these clade(s) needs to be confirmed by further studies containing a larger dataset of MLO proteins. Additional studies could be also addressed to the functional characterization of Rosaceae MLO homologs grouped in clade VII. Indeed, this appears to be basal to both clade IV and clade V (Figure 1), and thus might have contained ancestral proteins which later on evolved into PM susceptibility factors.

### Synteny between apple, peach and woodland strawberry *MLO*genes

We took advantage of recent developments in Rosaceae genomics in order to detect synteny between *P. persica*, *F. vesca* and *M. domestica* chromosomal regions containing *MLO* homologs. This permitted the inference of ortholgous relationships between *MLO* genes in these species. Notably, all predicted *MLO* orthologs from different Rosaceae species, fell in the same phylogenetic clade (Tables 1, 2 and 3; Figure 1 and Additional file 1). This is to be expected, since orthologs generally share the same function and thus are characterized by a high level of sequence conservation. It is noteworthy that the chromosomal localization of predicted *MLO* orthologs between *P. persica*, *M. domestica* and *F. vesca* is in accordance with the results of the synteny study performed after the release of the three genomes [33, 34]. In particular, genes situated on peach scaffold 2, 7 and 8 were predicted to have orthologs on strawberry chromosome 7, 1 and 2, respectively, whereas genes on peach scaffold 4 were predicted to have orthologs on strawberry chromosomes 2 or 3 (Table 4). *FvMLO3* was predicted to be orthologous to two peach *MLO* genes*, PpMLO8* and *PpMLO18*, which are localised in close proximity to each other on peach scaffold 6 and grouped together in clade VI. In this case, we hypothesize a relation of co-orthology due to the occurrence of a recent tandem duplication event in the peach genome. Similarly, *PpMLO5* and *FvMLO16* were predicted to be orthologs of two apple *MLO* genes, *MdMLO10* and *MdMLO17*, located on chromosomes 3 and 11. This is consistent with indications of duplications of large segments of these two chromosomes during the evolution of the apple genome [32].

### Transcription of apple putative *MLO*genes in response to *P. leucotricha*inoculation

In barley, pea and tomato, only one of the clade V *MLO* homologs seems to be involved in powdery mildew susceptibility, whereas in *A. thaliana* three *MLO* genes in Clade V are required to be inactivated in order to achieve a fully resistant phenotype [16, 27]. This implies that, within Clade V *MLO* genes, a further selection might be required to identify PM susceptibility genes. Accumulating evidence indicates that *MLO* susceptibility genes are up-regulated upon challenge with powdery mildew fungi [17]. Therefore, we analysed the expression level of apple *MLO* genes identified in this study in response to the interaction with *P. leucotricha*. Three pathogen-dependent gene up-regulations were detected. Two up-regulated *MLO* homologs, *MdMLO11* and *MdMLO19*, encode for proteins falling in clade V, thus making them likely candidates to act as PM susceptibility genes in apple. *MdMLO11* and *MdMLO19* are located on chromosomes 4 and 12 respectively, and are therefore both generated from a duplication event in the 9-chromosome ancestor of apple [32]. A third pathogen-dependent up-regulated gene, *MdMLO18*, was found, which encodes a protein grouping in the newly identified Clade VII (Figure 1). The presence of a PM upregulated gene outside clade V is consistent with transcriptome analyses recently performed in tomato (Appiano *et al*., unpublished results). Apple clade V also contains two genes, *MdMLO5* and *MdMLO7*, which show no significant changes in expression following inoculation. Accordingly, the lack of up-regulation of some clade V *MLO* genes has been observed in grapevine and tomato [23, 24]; Appiano *et al*., unpublished results). The possible role of these genes as susceptibility factors has not yet been highlighted.

*PpMLO3*, *PaMLO3* and *FvMLO4* are likely to represent true orthologs of *MdMLO19* (Table 4). Since orthologs often maintain the same function during evolution, we conjecture that the expression of these genes might also be responsive to PM fungi attacking corresponding species. Moreover, *FvMLO15* and *PpMLO9* are likely orthologs of *MdMLO18*, so they should also be considered as putative transcriptionally responsive genes to PM fungi attack. Further studies aimed at the functional characterization of these genes (e.g. through the application of reverse genetic approaches of targeted mutagenesis or gene silencing), in apple but also in peach and strawberry, might lead to the identification of resistant phenotypes, which could be used for the development of PM resistant cultivars. Particularly, studies on *MdMLO18* could lead to the characterization of a possible role for clade VII in the interaction with PM fungi.

## Conclusions

Our work led to the identification of 19 *MLO* homologs in peach, 17 in strawberry and 21 in apple. Three, three and four homologs, respectively, belong to clade V and therefore are candidates for being S-genes. Due to the high similarity between peach and apricot, we were able to amplify and characterize three Clade V apricot *MLO* genes.

The phylogenetic analysis revealed two new Rosaceae specific clades for the MLO family, although this needs to be confirmed by the use of a larger MLO proteins dataset.

Through inoculation of apple with *P. leucotrica*, we identified three up-regulated genes, i.e. *MdMLO11*, *MdMLO18* and *MdMLO19. MdMLO11* and *MdMLO19,* that belong to Clade V, are positioned in duplicated regions and have high sequence identity, therefore they are likely to be recent paralogs. *MdMLO18* belongs to the newly identified Clade VII.

## Methods

### *In silico*identification and comparison of MLO predicted proteins in peach, woodland strawberry and apple

Predicted peptides from the peach genome (v. 1.0) and the strawberry genome (v.1.0) gene model databases, available at the website of the Genomic Database for Rosaceae (http://www.rosaceae.org) [35], were queried for the presence of MLO homologs protein sequences. First, a BLAST search, using the tomato SlMLO1 amino acid sequence as query was carried out. A further search was performed with the HMMER programme, which uses a method for homolog searches based on the profile hidden Markov probabilistic model [36]. The sequences obtained with the previously mentioned BLAST search, were used together with other known *MLO* sequences from dicot and monocot species, namely: four RhMLOs from *Rosa hybrida*, 15 AtMLOs from *Arabidopsis thaliana*, SlMLO1 from *Solanum lycopersicum*, CaMLO2 from *Capsicum annuum*, PsMLO1 from *Pisum sativum*, MtMLO1 from *Medicago truncatula*, LjMLO1 from *Lotus japonicus*, VvMLO14 from *V. Vinifera*, HvMLO *from Hordeum vulgare*, TaMLO1_A1b and TaMLO_B1 from *Triticum aestivum* and OsMLO2 from *Oryza sativa*. MLO protein sequences from apple (*Malus domestica* cv. Golden Delicious) were identified by searching for the MLO domain profile (IPR004326) in the apple genome available at FEM-IASMA computational biology web resources (http://genomics.research.iasma.it). The resulting list was integrated with a BLAST search, carried out with the amino acid sequences previously listed for the HMMER search in peach and strawberry.

Chromosomal localization and predicted introns/exons structure of each *MLO* gene of apple, peach and strawberry was deduced based on the available genomic information at the GDR database. The presence and number of membrane spanning helices was predicted using the online software InterPro (http://www.ebi.ac.uk/interpro). Alignments for conserved amino-acids analysis were performed with the CLC Sequence Viewer v. 6.9 software (http://clcbio.com).

A total of 90 MLO protein sequences, including three apricot *MLO* sequences isolated *in vitro* (see next paragraph), were used for Clustal alignment (http://www.ebi.ac.uk/Tools/msa/clustalw2/). UPGMA-based and Neighbour-Joining-based phylogenetic trees were obtained with the CLC sequence viewer software. The UPGMA clustering algorithm was further used as input for the Dendroscope software, suitable for the visualization of large phylogenetic trees [37].

Relationships of orthology between *MLO* candidate genes from peach, woodland strawberry and apple were inferred by running the GBrowse-Syn tool available at GDR (http://www.rosaceae.org/gb/gbrowse_syn/peach_apple_strawberry) [35, 38]. This displays syntenic regions among the three available genomes of Rosaceae, as detected by the Mercator programme [35, 39]. For 50 Kb chromosomal stretches flanking each *P. persica PpMLO* homolog, syntenic regions from *F. vesca* and *M. domestica* were searched. Orthology was called upon the identification of *F. vesca* or *M. domestica MLO* homologs within syntenic blocks.

### *In vitro*isolation of apricot *MLO*homologs

RNA from apricot leaves (cultivar Orange Red) was extracted by using the SV Total RNA Isolation System Kit (Promega), and corresponding cDNA was synthesized by using the QuantiTect Reverse Transcription Kit (Qiagen) with oligo(dT) primers. Sequences of the peach *MLO* homologs *PpMLO1*, *PpMLO3* and *PpMLO4*, are phylogenetically close to *MLO* homologs functionally associated to PM susceptibility, and were therefore used to design the primer pairs 5 ′ -ATGGCAGCCGCAACCTCAGGAAGA-3 ′ /5 ′ -TTATATACTTTGCCTATTGTCAAAC-3 ′ , 5 ′ -ATGGCAGGGGGAAAAGAAGGACG-3 ′ /5 ′ -TCAACTCCTTTCTGATTTCTCAA-3 ′  and 5 ′ -ATGGCCGAACTAAGTAAAGA-3 ′ /5 ′ TCAACTTCTTGATTTTCCTTTGC-3 ′ , respectively. These were employed to amplify full-length cDNA sequences of apricot putative orthologs, by using the AccuPrime Taq polymerase (Invitrogen). Amplicons were purified by using the NucleoSpin Extract II kit (Macherey-Nagel) and ligated (molar ratio 1:1) into the pGEM-T Easy vector (Promega). Recombinant plasmids were cloned in *E. coli* DH10Î² chemically competent cells and recovered by using the Qiaprep spin miniprep kit (Qiagen). Sequencing reactions were performed twice, by using universal T7 and SP6 primers (Eurofins MWG Operon).

### Glasshouse test with apple cultivars

A total of 192 apple plants from three cultivars (Braeburn, Golden Delicious and Gala) were used to measure transcript abundance of *MLO* genes. Budwoods from these cultivars were grafted on M9 rootstocks in January 2012. The grafts were kept at −1°C for 2 months, and potted at the beginning of March in greenhouse. The plants grew for 6 weeks in the greenhouse at 20°C during the day, 17°C during the night, relative humidity of 70% and natural day/night cycle.

*P. leucothrica* was collected from apple trees in an unsprayed test orchard and used to infect greenhouse grown apple seedlings from ‘Gala Galaxy’ seeds. Four weeks after inoculation, conidia were used for the inoculation experiment, or transferred to new seedlings, to keep them viable. We inoculated by touching the plants with heavily infected apple seedlings. Control plants were not inoculated and kept separated in the same greenhouse of the inoculated plants. Inoculated and control plants were grown in the greenhouse at the growing conditions previously mentioned. The leaf samples were collected 4, 6, 8 and 24 hours post-inoculation (hpi).

Eight experimental repeats were performed and each sample contained three or four young leaves collected from each single plant. Every plant was used for sampling only once, to avoid any possible effect of wounding on the expression *of MLO* genes. The smallest statistical unit was a plant. The leaves were flash-frozen and ground in liquid nitrogen, and stored at −80°C until RNA extraction.

### qPCR analysis of transcript levels

RNA extraction was carried out with the MagMAX-96 Total RNA isolation kit (Applied Biosystem) that includes DNAse treatment. The kit yielded between 50 and 200 ng/ul, of good quality RNA per sample.

Primers for gene expression analysis were designed with NCBI Primer Designing Tool (http://www.ncbi.nlm.nih.gov/tools/primer-blast/). Four serial dilutions of cDNA (1/5 - 1/25  – 1/125  – 1/625) were used to calculate the efficiency of each primer pair with iCycler software (Biorad). In case of efficiency lower than 1.80 or greater than 2.20, the primer pair was discarded and a new one tested, with the exception of *MdMLO9*, for which was not possible to design a primer pair with better efficiency. It was only possible to analyse 19 *MLO* genes because for *MdMLO12* and *MdMLO16* was not possible to design specific and efficient primer pairs, despite numerous attempts. Presence of a specific final dissociation curve was determined after each qPCR amplification reaction with progressive increment of temperature from 65°C to 95°C (0.5°C each step, 5 sec) and the size of the product was confirmed by agarose gel electrophoresis.

Quantitative Real Time-PCR (qPCR) was performed with SYBR greenER mix (Invitrogen) in a 15-Î¼L reaction volume, using a Bio-Rad iCycler iQ detection system, run by the Bio-Rad iCycler iQ multicolor 3.1 software. The software applies comparative quantification with an adaptive baseline. Samples were run in two technical replicates with the following thermal cycling parameters: 95°C 3 min  – 95°C 15 sec, 60°C 1 min (repeated 40 times)  – 95°C 10 sec.

Reference genes Î²-actin (NCBI accession number DT002474; Plaza accession number MD00G171330 - http://bioinformatics.psb.ugent.be/plaza/), ubiquitin (Plaza accession number MD05G001920) and elongation factor 1 (Plaza accession number MD09G014760) were used as reference genes (Table 5). All these three genes were used in previous works [40–42]. For additional control, we assessed the stability of our genes with the software geNorm (medgen.ugent.be/~jvdesomp/genorm/). An M-value lower than 1.5 is generally considered as stable enough [43–45] and all three reference genes in all three cultivars considered are within this threshold. We saw differences in stability between cultivars: ‘Golden Delicious’ was the most stable cultivar (actin: 0.824  – ubiquitin: 0,852  – elongation factor 1: 0,926), whereas ‘Braeburn’ was the less stable (actin: 1.246  – ubiquitin: 1,293  – elongation factor 1: 1,369) and ‘Gala’ showed intermediate stability (actin: 1.039  – ubiquitin: 1,152  – elongation factor 1: 1,078).

**Table 5:** **Gene-specific primers and amplicon sizes in qRT-PCR detection of 19** ***MdMLO*** **-like genes based on** ***Malus domestica*** **cv. Golden Delicious genome sequence**

Gene	Forward primer (5 ′ – 3 ′ )	Reverse primer (5 ′ – 3 ′ )	Size (bp)	Efficiency
*MdMLO1*	GTGGGCTCGGTCGGCCAAAA	CCAGCACCAGCACCAGAACCA	81	2.06
*MdMLO2*	CGTTGGATCAACCACTGCGCCT	TGAGCTGCAGCCAGTGGGATCT	87	1.83
*MdMLO3*	CCACTGCGCCTCTCTGAAGCA	CCACCAAAACGGCTCTCCAGGT	93	2.12
*MdMLO4*	TGTTGCAGACACTATGCTGCCATGT	GGCAGCAGCTAAAGATCTGCGT	109	1.87
*MdMLO5*	TCGTCAGGCTCTCATTCGGGGT	GTGCTGCTGCCACTCCCTC	132	1.80
*MdMLO6*	TTCGCGGAGGAGGGGTCGTT	TTCGAGCGACAGCAACGGCA	72	2.15
*MdMLO7*	TGGAGCAAGTCACCAGTCTCCAT	CGCTTCCTGGTGCCAAATGTGC	127	2.12
*MdMLO8*	GTCAAGCTAATCTTACCACGCGCT	GGCTGGAAGGAAGGACAGCCA	85	1.95
*MdMLO9*	GCTGCAACACGTAATCACCC	AGAACGCCATTTCGAAAGCA	173	2.30
*MdMLO10*	GCGATCGTTGGCCTTGACTCC	TTCCGCGCTCGACAAGCAGA	86	1.92
*MdMLO11*	CCGTTCCATCACCAAGACGA	ATTGCTCTCCGAGTTACGCC	102	1.90
*MdMLO13*	ACATTGTCCCCAGGCTTGTT	GCCCAACCAATAAGTCCCGA	151	2.00
*MdMLO14*	TGCACTTGTCAGCCAGATGGG	GCATCTCCCACCCACGAACCG	81	2.15
*MdMLO15*	GCGCCTTTCTCTCTGCTGGGT	CGCGTGCGAGGTGGTCTCTT	90	2.01
*MdMLO17*	TTGCCACTGTATGCCTTGGT	TGCTTGCTTCTGTGCGAATG	163	2.15
*MdMLO18*	AAGGAAGGCTCTCATTCAGGCTCT	TGCAATTGGCTTTTGACCAACGGT	100	2.22
*MdMLO19*	CAGAGTGGCGACTGCACTTA	GGGACATGGAGTGCAAAGGA	110	1.97
*MdMLO20*	AAAAAGCTCCACCAACCCCA	TTTCTCTCCCATGACGCTCG	165	2.11
*MdMLO21*	CCTTGTTCGAGGCCGTAGAG	ACCAAGTGCTTTGGTGGTTT	176	1.95
Î²-actin	CTATGTTCCCTGGTATTGCAGACC	GCCACAACCTTGATTTTCATGC	82	1.90
Ubiquitin	CATCCCCCCAGACCAGCAGA	ACCACGGAGACGAAGCACCAA	349	1.91
Elongation Factor 1	TACTGGAACATCACAGGCTGAC	TGGACCTCTCAATCATGTTGTC	308	2.07

Each of the biological replicates was analysed in duplicate and the average of these two replicates was used for further analysis. In case of excessive difference between the two replicates (one Ct or more), the run was repeated. Considering the high number of samples and genes of interest, we opted for this approach in order to reduce the number of total runs. Data analysis was performed according to Hellemans *et al.* [46], using the statistical package SPSS (IBM). This analysis method takes into account the efficiency value of each primer pair. For some genes it was necessary to apply a natural log transformation to the data, in order to obtain normal distribution of residues. To investigate the differences between control and inoculated samples, we used *T*-test (p ≤ 0.05).

## Availability of supporting data

The following files are available on: mynotebook.labarchives.com.Figure 1  – Phylogenetic tree of Rosaceae MLO.

**DOI:** 10.6070/H4Z60M0N.

Additional file 1 - Synteny between apple, peach and strawberry.

**DOI:** 10.6070/H4TD9V8C.

## Electronic supplementary material

Additional file 1: **Synteny between apple, peach and strawberry.** Results of search for *F. vesca* and *M. domestica* regions syntenic to 50 kb *P. persica* chromosomal stretches containing the *PpMLO* homologs identified in this study. Shaded polygons indicate aligned regions between genomes. Grid lines are drawn to indicate insertions/deletions between the genomes of *F. vesca* and *M. domestica* with respect to the *P. persica* reference sequence. *P. persica*, *F. vesca* and *M. domestica MLO* homologs, named according to the nomenclature of the Genomic Database of Rosaceae, are boxed. (PDF 817 KB)

Additional file 2: **Transcriptional variation of 19 apple MLO genes in three cultivars following inoculation with** ***P. leucotricha.*** Transcription abundances of 19 *MLO*-like genes following powdery mildew (PM) inoculation in ‘Golden Delicious’ (1a), ‘Gala’ (1b) and ‘Braeburn (1c) leaf samples. The graphs show expression values of inoculated samples relative to control samples, averaged from four to eight biological replicate, normalized, that are in turn the average of two experimental replicates. The Ct values have been normalized with three reference genes: actin, ubiquitin and elongation factor 1. Statistical significance was determined with a *t*-test for each individual pair of inoculated and control samples at each time point (4, 6, 8 and 24 hpi). The error bars show standard errors of the means. Significant differences between inoculated samples and control samples are indicated with a *(*P* < 0.05). (PDF 1 MB)
