# Supplementary figures and images for: Characterization of the MLO gene family in Rosaceae and gene expression analysis in Malus domestica
Source: BMC Genomics. 2014 Jul 22;15(1):618. doi: 10.1186/1471-2164-15-618 (PMC4124139; doi:10.1186/1471-2164-15-618)

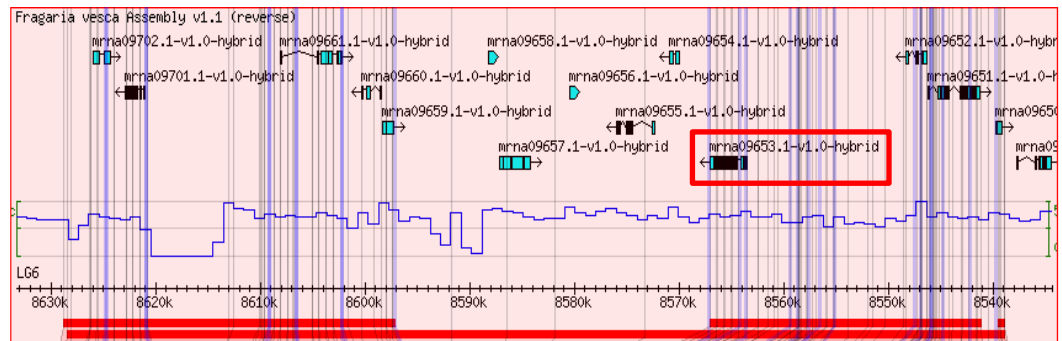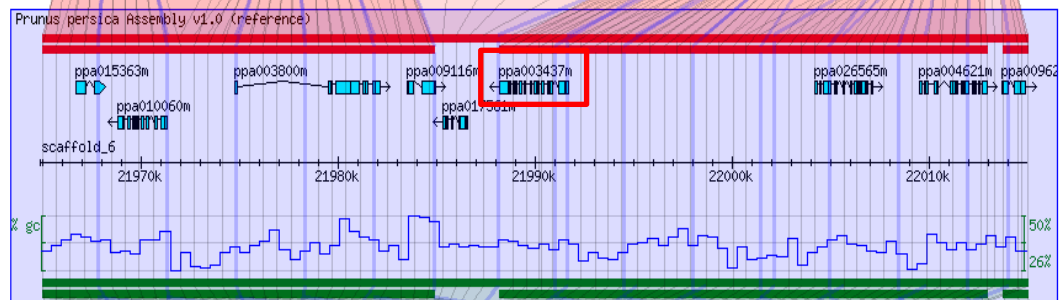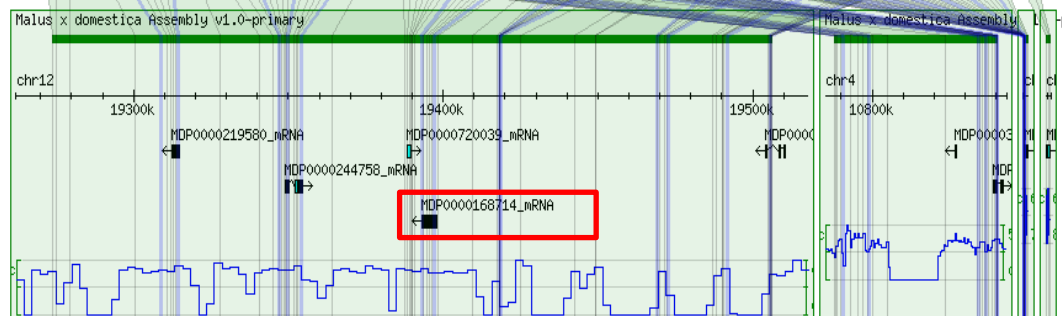

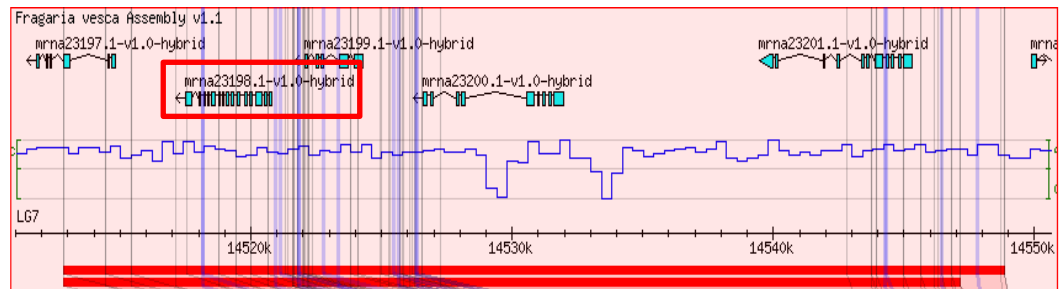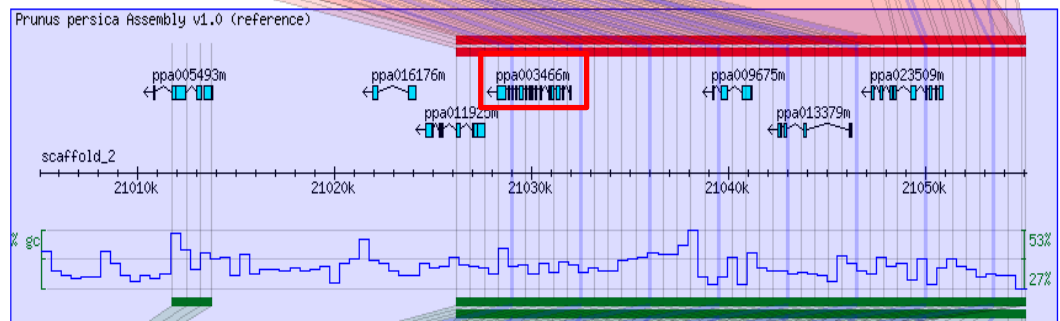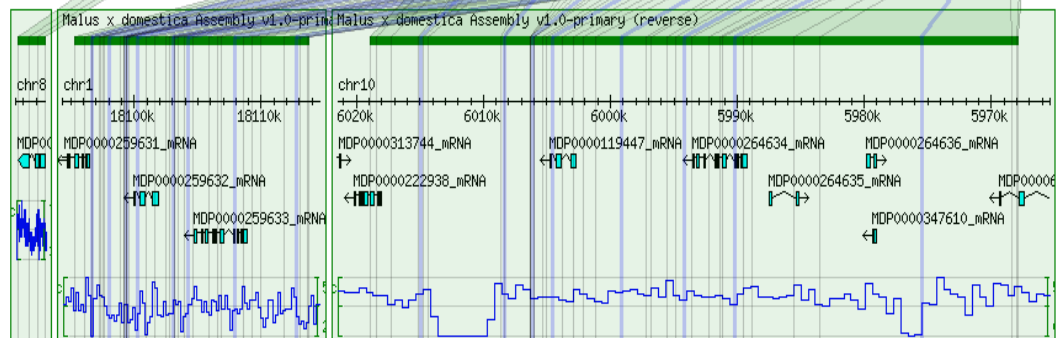

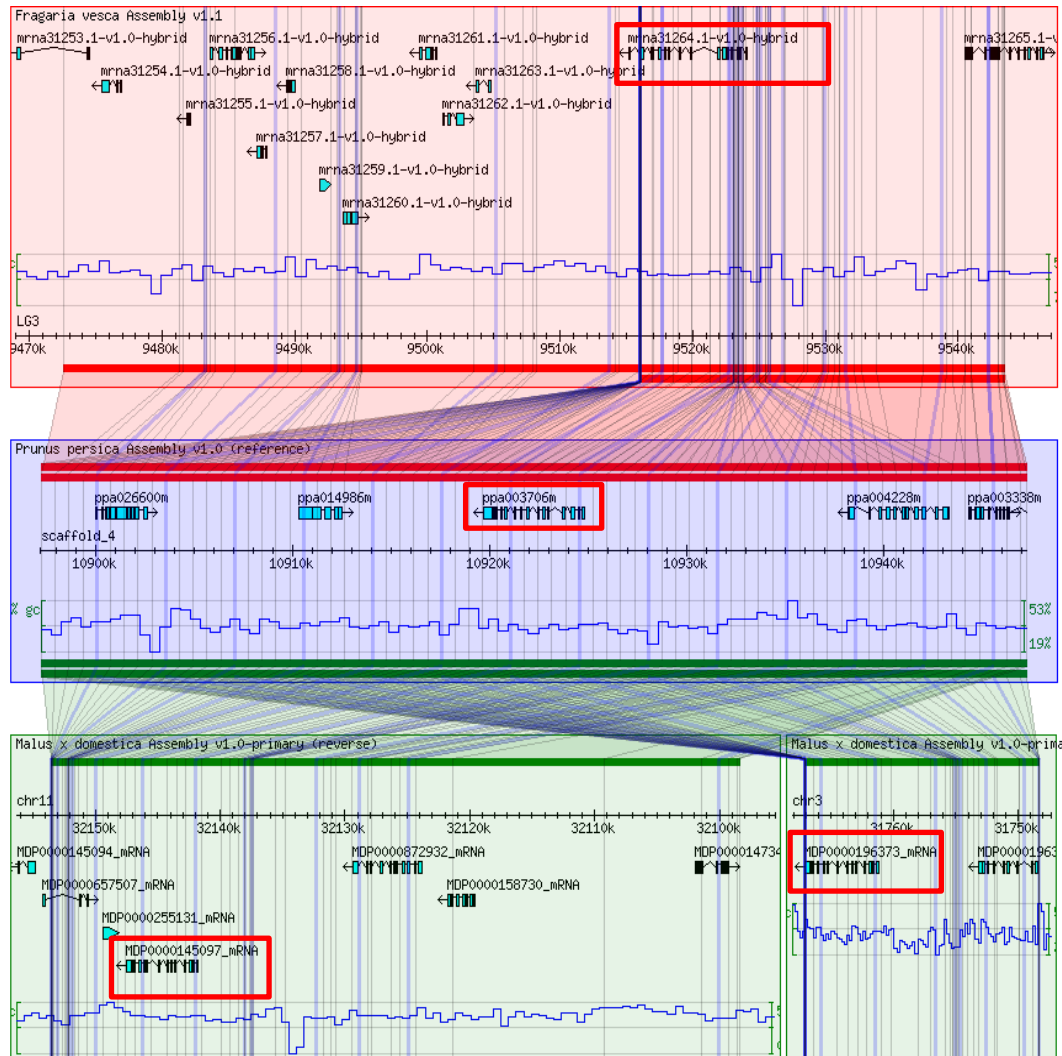



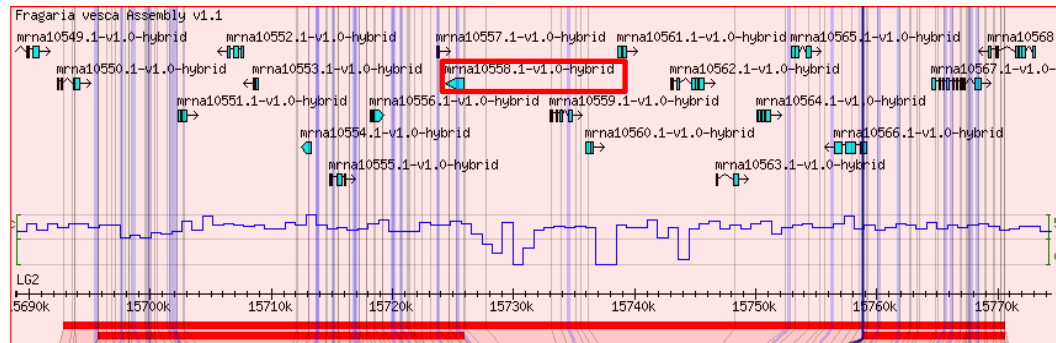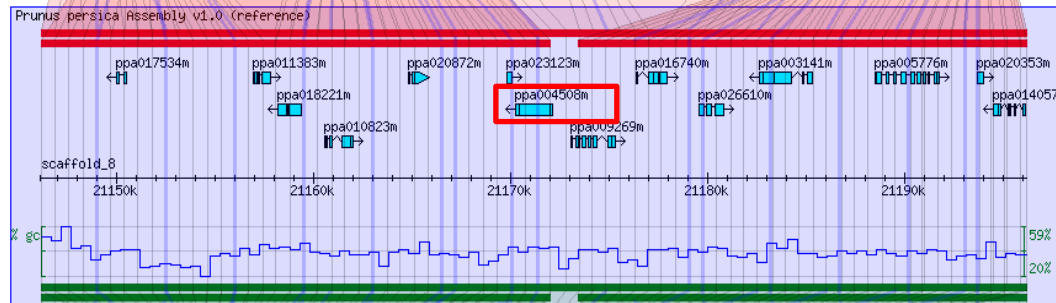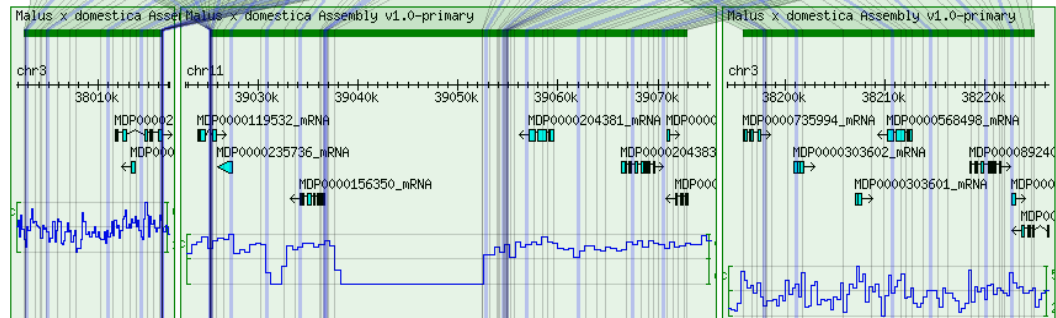

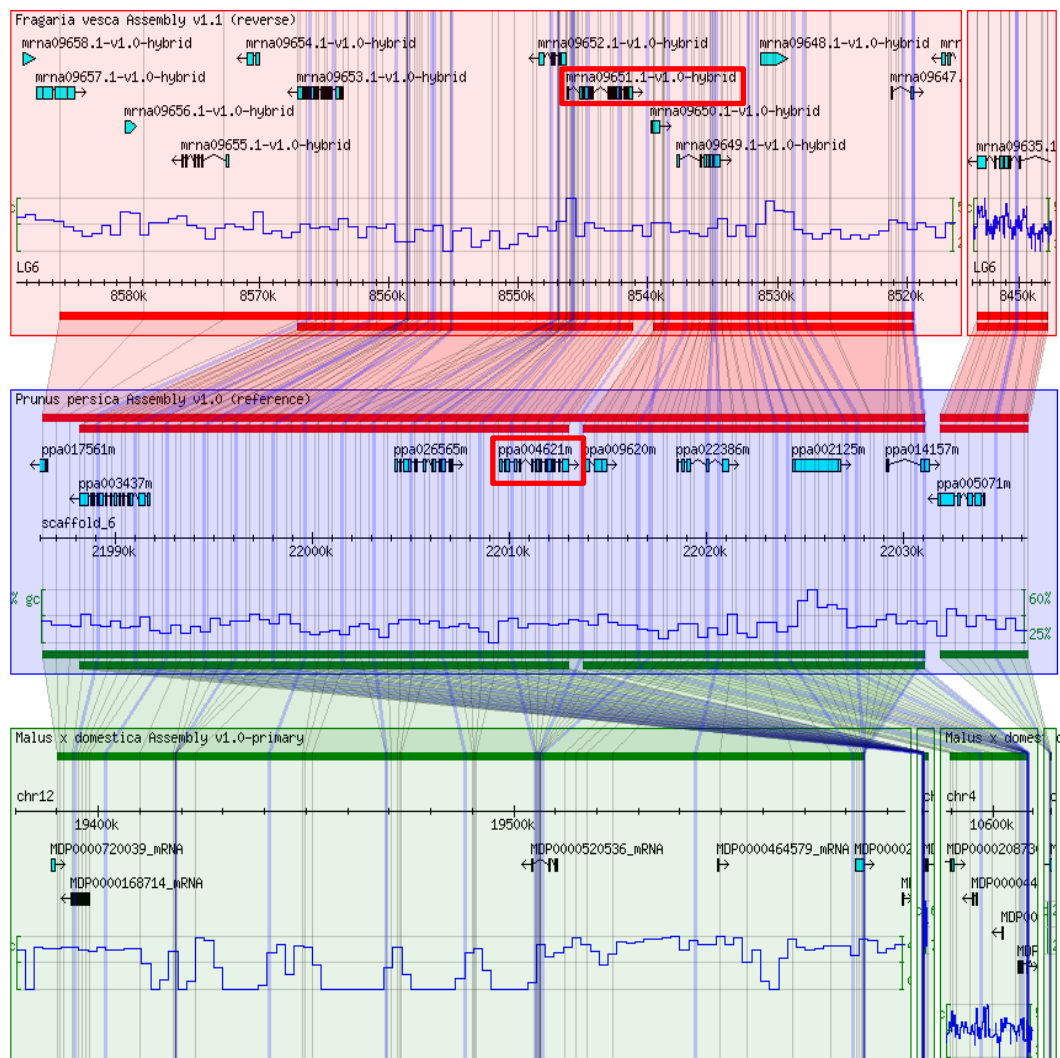

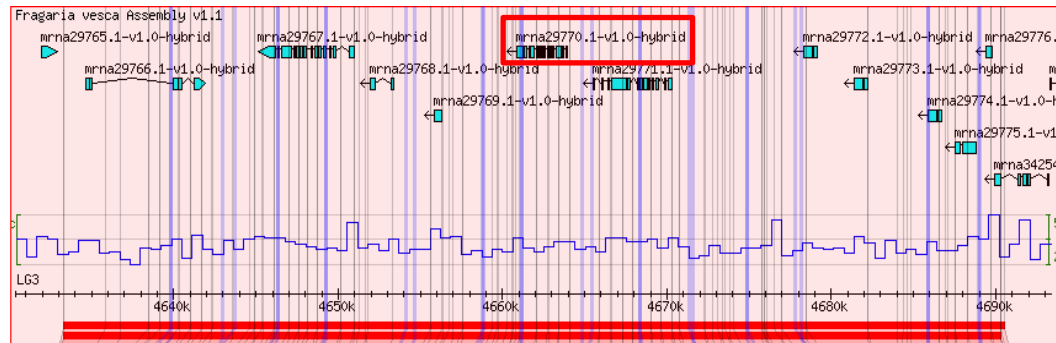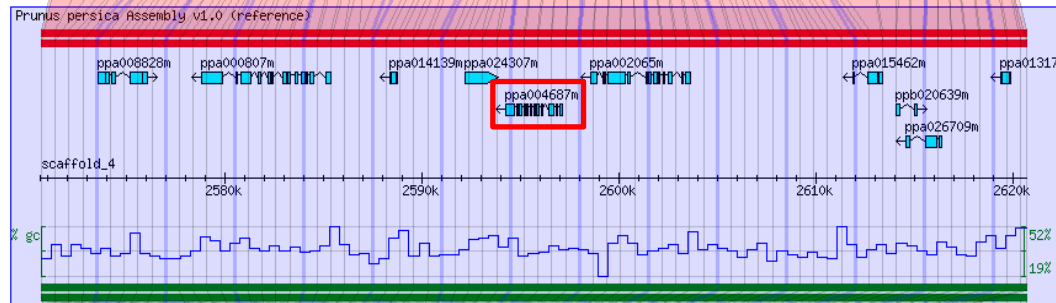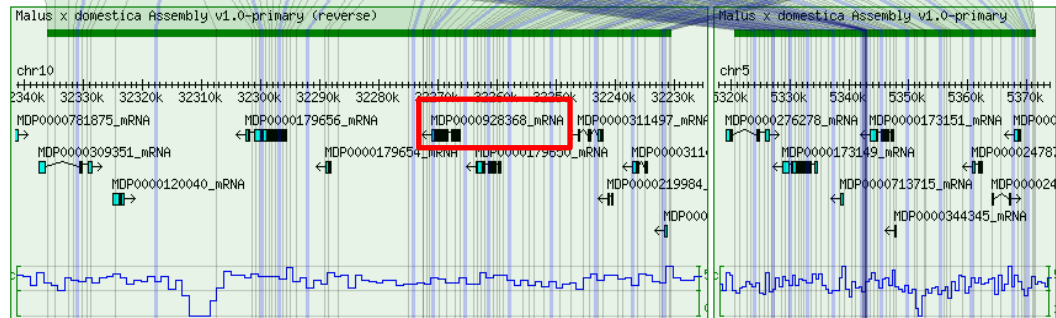

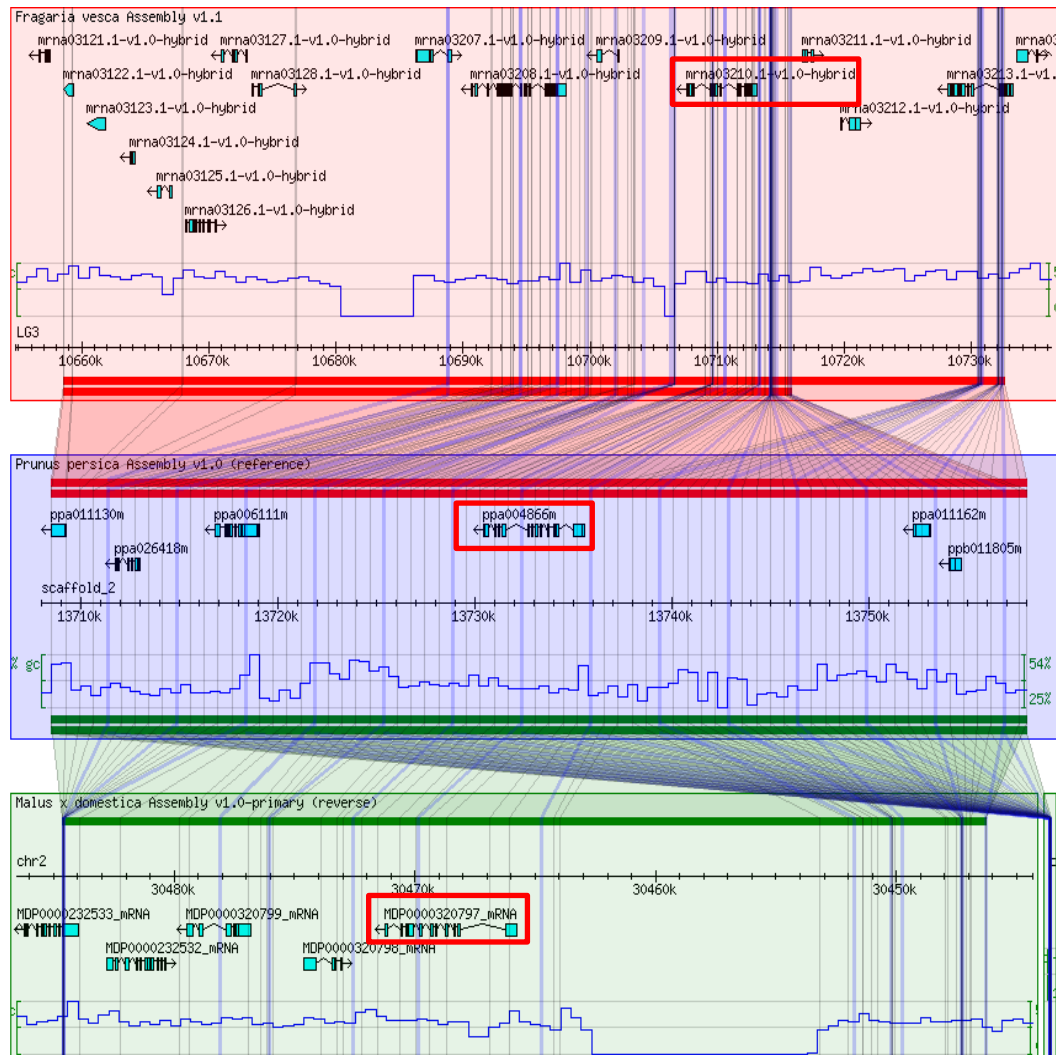

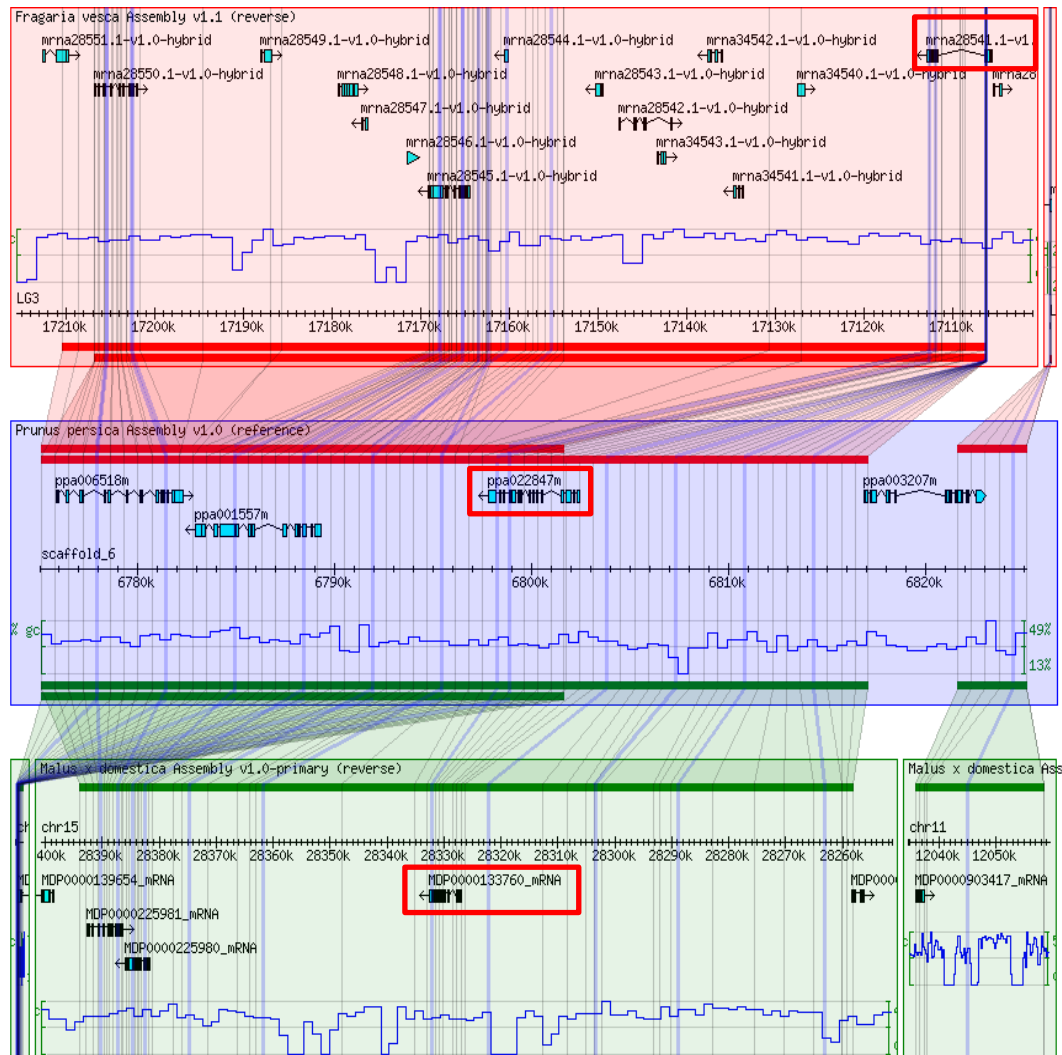

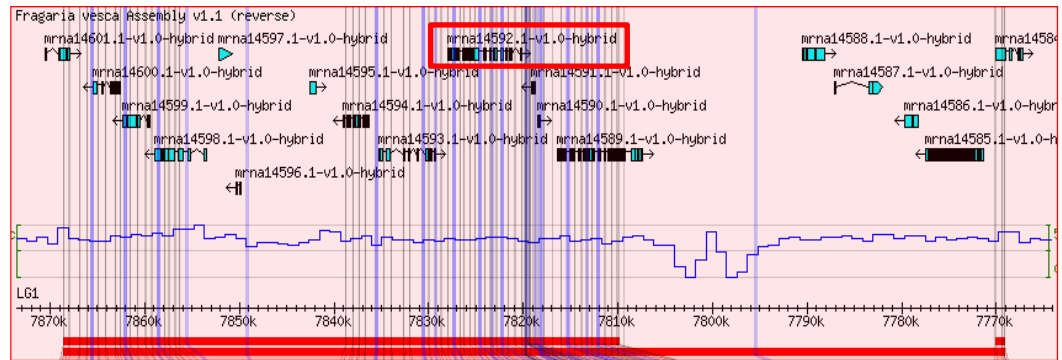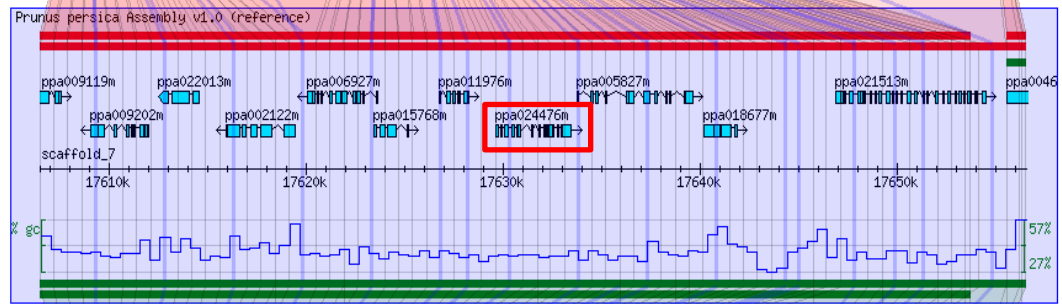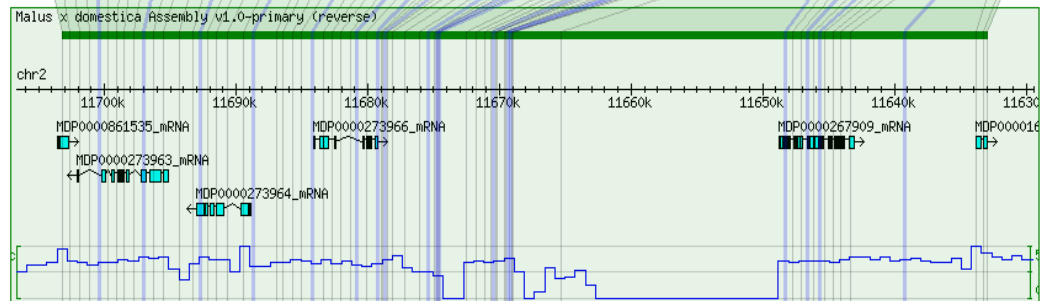

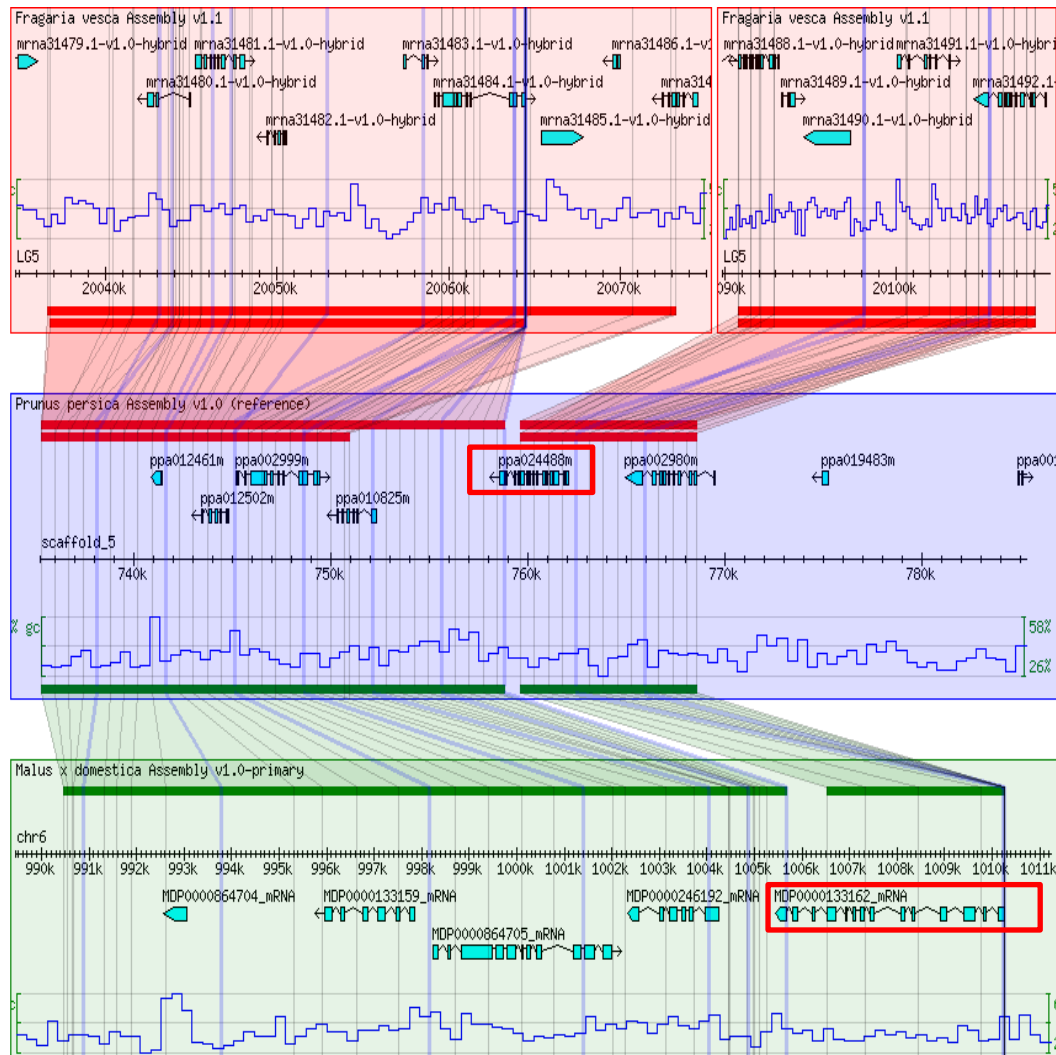

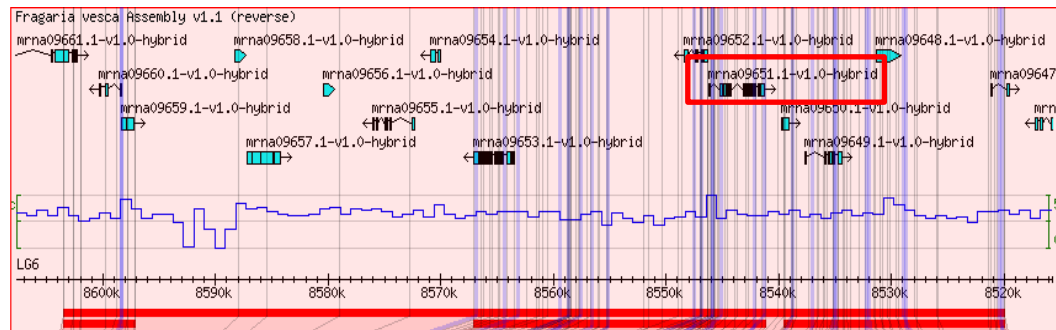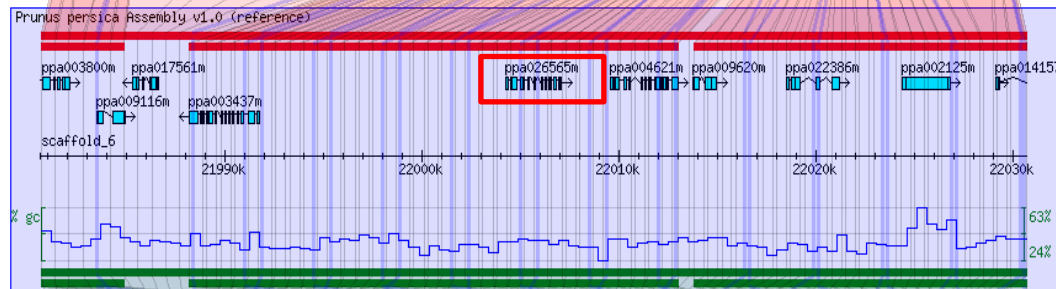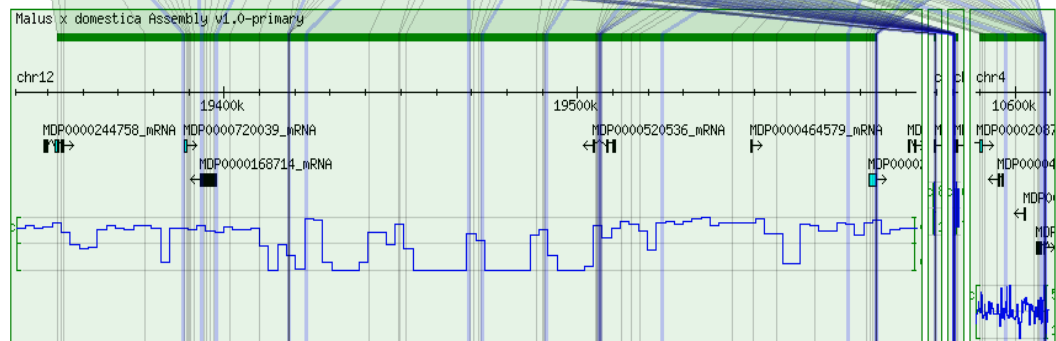

Supplement: Supplementary file 1 — Additional file 1: Synteny between apple, peach and strawberry. Results of search for F. vesca and M. domestica regions syntenic to 50 kb P. persica chromosomal stretches containing the PpMLO homologs identified in this study. Shaded polygons indicate aligned regions between genomes. Grid lines are drawn to indicate insertions/deletions between the genomes of F. vesca and M. domestica with respect to the P. persica reference sequence. P. persica, F. vesca and M. domestica MLO homologs, named according to the nomenclature of the Genomic Database of Rosaceae, are boxed. (PDF 817 KB) [file 12864_2014_6323_MOESM1_ESM.pdf]

# A. GOLDEN DELICIOUS

Transcript fold change

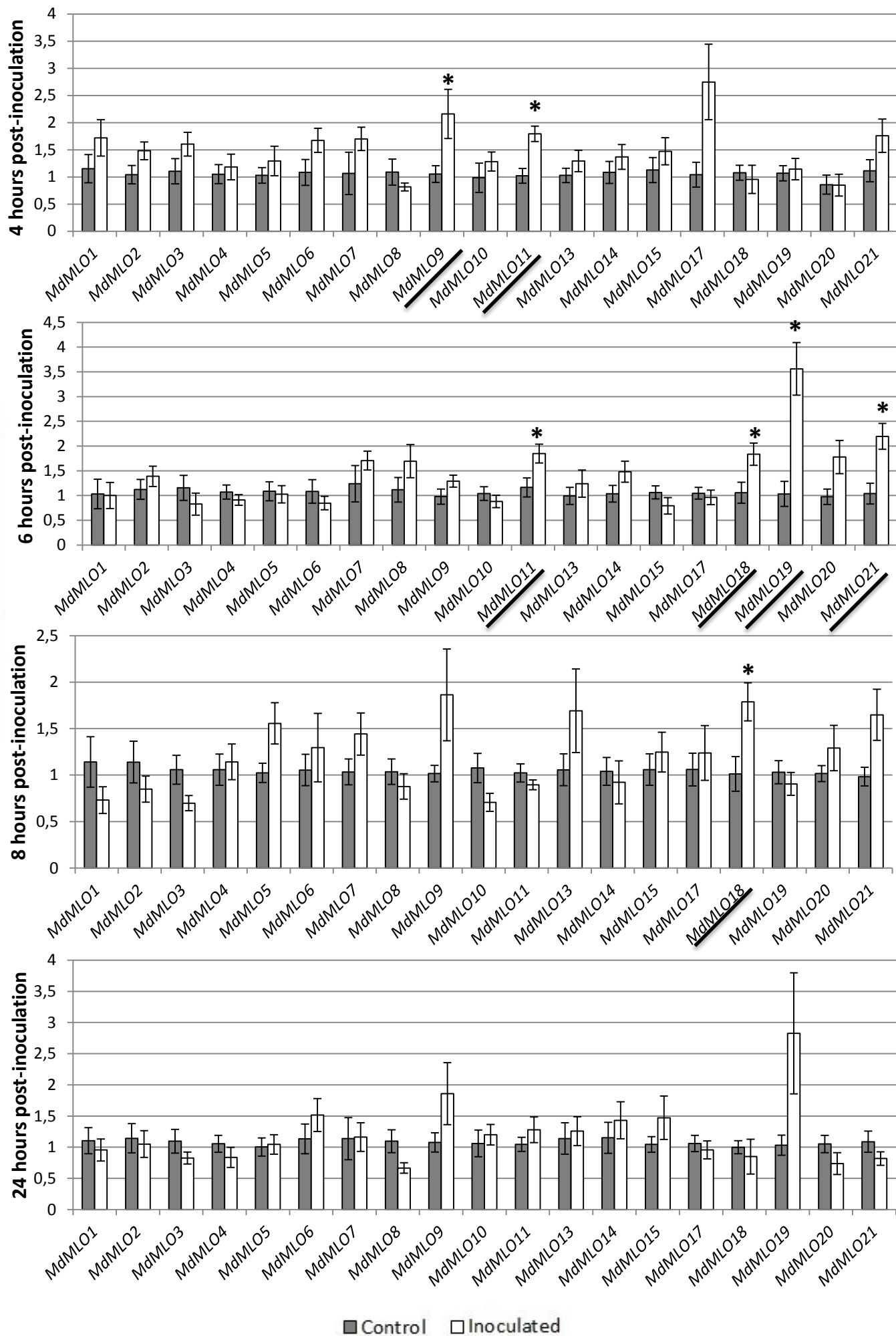

B. GALA

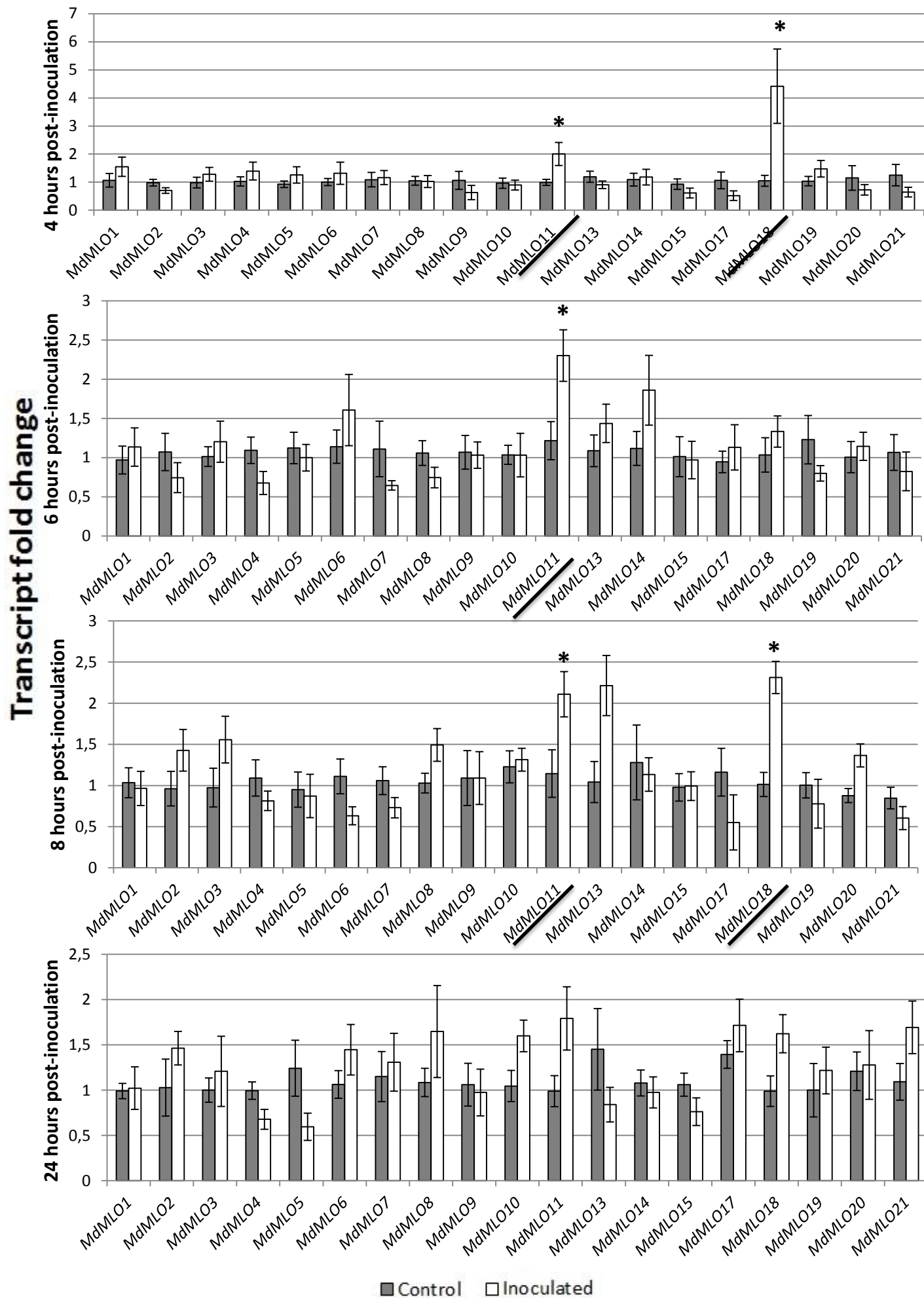

C. BRAEBURN

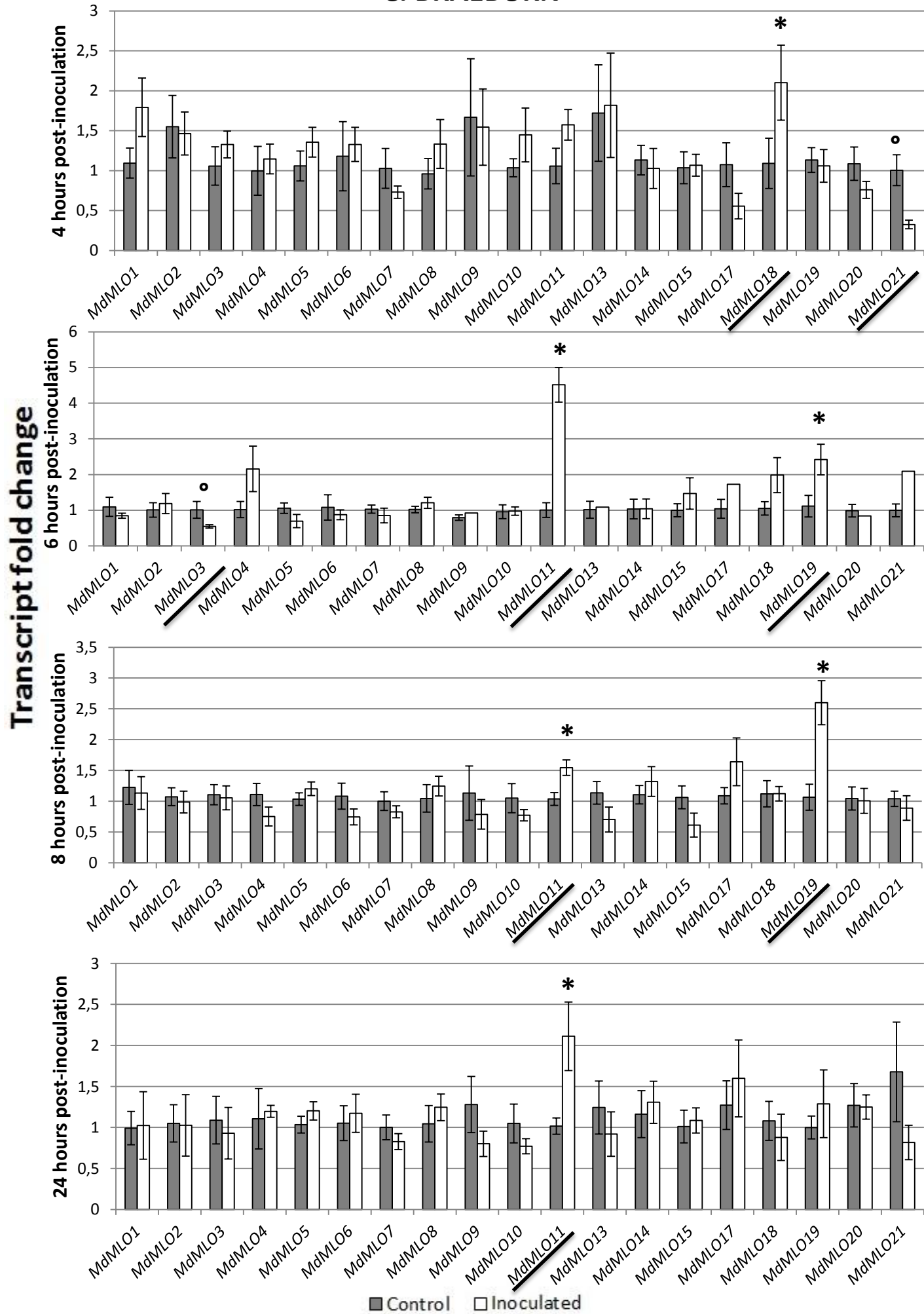

Supplement: Supplementary file 2 — Additional file 2: Transcriptional variation of 19 apple MLO genes in three cultivars following inoculation with P. leucotricha. Transcription abundances of 19 MLO-like genes following powdery mildew (PM) inoculation in ‘Golden Delicious’ (1a), ‘Gala’ (1b) and ‘Braeburn (1c) leaf samples. The graphs show expression values of inoculated samples relative to control samples, averaged from four to eight biological replicate, normalized, that are in turn the average of two experimental replicates. The Ct values have been normalized with three reference genes: actin, ubiquitin and elongation factor 1. Statistical significance was determined with a t-test for each individual pair of inoculated and control samples at each time point (4, 6, 8 and 24 hpi). The error bars show standard errors of the means. Significant differences between inoculated samples and control samples are indicated with a *(P < 0.05). (PDF 1 MB) [file 12864_2014_6323_MOESM2_ESM.pdf]
